# Responses of the Lipoxygenase Gene Family to Drought Stress in Broomcorn Millet (*Panicum miliaceum* L.)

**DOI:** 10.3390/genes16040368

**Published:** 2025-03-23

**Authors:** Lin Cong, Lin Deng, Hongfei Yao, Yaoyuan Zhang, Hongying Li, Haigang Wang, Bin Zhang, Yuanhuai Han, Junjie Wang

**Affiliations:** 1 College of Agriculture, Shanxi Agricultural University, Taigu 030801, China; 18714507543@163.com (L.C.); linglindeng@163.com (L.D.); 20233158@stu.sxau.edu.cn (H.Y.); zhang3576285yy@163.com (Y.Z.); hongying1964@163.com (H.L.); zb0304209@163.com (B.Z.); 2 Center for Agricultural Genetic Resources Research, Shanxi Agricultural University, Taiyuan 030031, China; nkywhg@126.com; 3 Houji Laboratory in Shanxi Province, Taiyuan 030031, China

**Keywords:** broomcorn millet, *LOX* gene family, drought stress, gene expression, abiotic stress adaptation

## Abstract

**Background:** Broomcorn millet (*Panicum miliaceum* L.), a drought-tolerant C4 crop, is crucial for agricultural resilience in arid regions. Lipoxygenases (LOXs), key enzymes in plant stress responses, have not been studied in broomcorn millet. This study aimed to identify *LOX* genes in broomcorn millet and elucidate their role in drought tolerance. **Methods:** We employed bioinformatics and physiological analyses to identify *LOX* genes in broomcorn millet. Expression profiles were assessed in different organs, and drought stress responses were evaluated in tolerant (HSZ, YXDHM) and sensitive (YS10) varieties. Antioxidant enzyme activities (SOD, POD, CAT) and malondialdehyde (MDA) levels were measured. **Results:** Twelve *LOX* genes were identified, classified into three subfamilies, and mapped across seven chromosomes. These genes contained stress-responsive cis-elements and showed organ-specific expression, with *PmLOX5* exhibiting no detectable expression. Under drought stress, tolerant varieties showed elevated antioxidant activities and reduced MDA accumulation. *PmLOX2*, a homolog of Arabidopsis *AtLOX1*/*AtLOX5*, was significantly induced in tolerant varieties, correlating with enhanced antioxidant capacity and reduced oxidative damage. **Conclusions:** *PmLOX* genes, particularly *PmLOX2*, play a pivotal role in drought tolerance by modulating ROS scavenging and membrane protection. This study provides a foundation for leveraging *LOX* genes to improve drought resilience in broomcorn millet and related crops.

## 1. Introduction

Global climate change has made drought a important factor that affects crop yield and quality in many regions. As an abiotic stressor, drought significantly hinders crop growth and development, and negatively affects productivity [1]. It disrupts the osmotic regulation system in plants, causing an imbalance in reactive oxygen species (ROS) metabolism within cell membranes. This imbalance can lead to excessive ROS accumulation, causing lipid peroxidation and cell membrane damage. Additionally, protein denaturation and inactivation disrupt key physiological and biochemical processes, weakens photosynthesis, inhibits growth, and increases plant mortality [2,3,4]. Consequently, developing high-yield, high-quality, and drought-resistant crop varieties coupled with water-saving cultivation practices has become a pressing challenge in agriculture owing to limited water resources.

Broomcorn millet (*Panicum miliaceum* L.) is a short-day C4 crop belonging to the Gramineae genus, with a cultivation history in China dating back to 10,000 years [5]. Owing to its strong stress resistance, adaptability, drought tolerance, high water-use efficiency, short growth cycle, and alignment with the rainy season in arid and semi-arid regions, broomcorn millet thrives in various soils, particularly in dryland farming areas of the Loess Plateau. Its regional and production advantages make it a significant crop for disaster relief [6,7,8].

LOXs are oxidoreductases with non-heme iron as their active center and are widely distributed in both animals and plants [9]. In plants, LOXs contribute significantly to in physiological and biochemical processes and are present in various organs, including roots, stems, leaves, flowers, and fruits. As the key enzymes in the plant fatty acid metabolic pathway (LOX pathway), LOXs can significantly influence organ growth, development, fruit ripening, oil oxidation, and signal transduction processes related to abiotic and biotic stresses [10,11]. Moreover, LOXs regulate plant secondary metabolism, affecting the production of important compounds such as fruit flavors and flower aromas [12,13,14].

In plants, LOXs can catalyze the oxidation of linoleic acid (LA) and linolenic acid (LnA) at the C9 and C1 positions of the fatty acid carbon chain, respectively. LOXs can be categorized into two subfamilies according on the location of the bound carbon atom: 9-LOX and 13-LOX [15]. Based on similarities in sequence structure and whether chloroplast transit peptides are present, plant LOXs are categorized into type I and type II. Type I LOXs can share 5% sequence similarity and lack chloroplast transit peptides, while type II LOXs have lower sequence similarity but contain these peptides [16]. Currently, all 9-LOXs are type I, whereas 13-LOXs are classified into type I and type II subtypes [17].

Plant LOXs catalyze the production of hydroperoxides (HPOs), which serve as precursors for various bioactive substances through multiple downstream pathways. Jasmonates (JAs) synthesized via allene oxide synthase (AOS) and green leaf volatiles (GLVs) formed by hydroperoxide lyases (HPLs) play significant roles in plant responses to abiotic and biotic stressors [18,19]. GLVs contribute to plant defense by inducing the expression of defense-related genes and directly or indirectly suppressing pathogens and pests [20]. Similarly, JAs are major stress-signaling molecules, mediating plant responses to challenges, such as drought, salt stress, and pathogen invasion [21,22].

The *LOX* gene family has been identified and analyzed in several crops, including Arabidopsis (6 members), rice (14 members) [23], foxtail millet (12 members) [24], tomato (14 members) [25], and grape (18 members) [26]. In Arabidopsis, *AtLOX2* is involved in JA biosynthesis [27], whereas *AtLOX5* is essential for the development of lateral roots in plants and for resisting pathogens [28]. In rice, the overexpression and knockout studies have indicated that moderate inhibition of *OsLOX2* under normal storage conditions can slow seed deterioration without affecting germination [29]. In foxtail millet, the expression of *SiLOX7* increased in drought-resistant varieties under drought stress [24]. Despite these attributes, only a few drought-related gene families, such as YABBY [30], ASR [31], bZIP [32], and NAC [33], have been studied in broomcorn millet. However, the *LOX* gene family in this crop remains unexplored.

The study of LOXs in plants has provided valuable insights into their roles in various physiological processes and stress responses. However, the *LOX* gene family in broomcorn millet, a drought-tolerant C4 crop with significant agricultural importance in arid regions, remains largely unexplored. This study aims to fill this gap by systematically identifying and characterizing the *LOX* gene family in broomcorn millet. We investigate their evolutionary relationships, stress-responsive regulatory elements, and functional roles in drought tolerance through bioinformatic and physiological analyses. Our research contributes to a deeper understanding of the genetic basis of drought resistance in broomcorn millet and potentially facilitates the development of improved cultivars with enhanced drought resilience. We hypothesize that the *LOX* gene family in broomcorn millet plays a significant role in modulating drought tolerance by regulating ROS scavenging, membrane protection, and jasmonate signaling pathways.

## 2. Material and Methods

### 2.1. Identification and Retrieval of Broomcorn Millet LOX Family Member Sequences

Complete genome information, CDS sequences, protein sequences, and annotation files of broomcorn millet were obtained from the NCBI database [34]. Six AtLOX protein sequences from Arabidopsis were retrieved from the TAIR database [35], whereas twelve SiLOX protein sequences of foxtail millet were retrieved from the Phytozome v13 database [36]. Additionally, thirteen OsLOX protein sequences were downloaded from the National Rice Data Center. Using TBtools v2.152 software [37], a local BLAST (https://blast.ncbi.nlm.nih.gov/Blast.cgi, accessed on 25 February 2025) search was performed with broomcorn millet protein sequences as the database and the six *AtLOX* sequences as the queries, setting an E-value threshold of <1 × 10^−5^ and removing duplicate genes to preliminarily identify the *LOX* gene family members of the broomcorn millet. The SMART online database was used to identify the structural domains of candidate PmLOX proteins. Sequences containing only the lipoxygenase or PLAT/LH2 domain were excluded, resulting in the final determination of broomcorn millet *LOX* gene family members.

Chromosomal location information of the broomcorn millet *LOX* gene family members was extracted from the genome annotation file using the Sequence Toolkit option in TBtools. Subsequently, Gene Location Visualization from the GTF/GFF plugin in TBtools was utilized to construct a chromosomal location map.

### 2.2. Prediction of Amino Acids and Physicochemical Properties of LOX Family Members in Broomcorn Millet

The physicochemical properties of the *LOX* family of proteins in broomcorn millet, including the amino acid count, the isoelectric point, the molecular weight, and other characteristics, were analyzed and predicted using the ExPASy-ProtParam online tool [38].

### 2.3. Multiple Sequence Alignment and Phylogenetic Tree Construction

Multiple sequence alignments of the amino acid sequences of 12 *SiLOX* proteins from foxtail millet, 12 *OsLOX* proteins from rice, and 6 *AtLOX* proteins from Arabidopsis were performed using the MUSCLE program in MEGA7.0. A phylogenetic evolutionary tree (NJ tree) was constructed from the aligned sequences using the neighbor-joining method in MEGA7.0, with the model set to p-distance, bootstrap value set to 1000, and default settings for other parameters. iTOL was used to edit and visually enhance the resulting phylogenetic tree.

### 2.4. Prediction and Analysis of Conserved Motifs, Protein Secondary Structure, and Subcellular Localization

The MEME online tool was used to analyze and predict conserved motifs within the amino acid sequences of broomcorn millet LOX proteins [39]. TBtools was used for visualization [37]. WebLogo was used to create sequence logos for the conserved domains. The secondary structure of broomcorn millet *LOX* proteins was predicted with the SOPMA online tool. The subcellular localization of the identified PmLOX proteins was predicted using the Plant-mPLoc online platform.

### 2.5. Analysis of Cis-Acting Components in the Promoter Region of LOX Family in Broomcorn Millet

The 2000 bp promoter sequences upstream of the 12 *LOX* genes were extracted from the genome annotation information of broomcorn millet using the TBtools software. These sequences were submitted to the PlantCARE online platform for *cis*-acting element predictions.

### 2.6. Plant Growth Conditions

Three kinds of broomcorn millet were used as varieties with varying drought tolerances in this study: drought-resistant varieties HSZ and YXDHM and drought-sensitive variety YS10 after evaluation [40]. Seeds of three broomcorn millet varieties (HSZ, YXDHM, YS10) were planted in pots with a 2:1 nutrient soil-to-vermiculite ratio. Plants were grown in an artificial climate chamber of at the Agricultural College of Shanxi Agricultural University under controlled conditions: light intensity 14,000 lx, temperature 28 °C/22 °C (day/night), 16 h/8 h light/dark cycle, and 80% humidity. Seedlings were maintained until the four-leaf stage before drought treatment.

### 2.7. Drought Stress Treatment

Drought stress was imposed on four-leaf-stage broomcorn millet seedlings by completely withholding irrigation to simulate natural drought conditions. Control plants were maintained under well-watered conditions throughout the experiment. Soil water content (*SWC*) was monitored gravimetrically using the formula:

$$SWC(\%)=\left(\frac{Fresh\;weight-Dry\;weight}{Dry\;weight}\right)\times 100$$

where Fresh weight and Dry weight represent the mass of soil samples before and after oven-drying at 105 °C for 48 h, respectively. Drought stress initiation was defined when *SWC* dropped below 30% of field capacity (FC), reflecting moderate-to-severe drought conditions in arid agroecosystems.

The penultimate leaves of aboveground seedlings were collected at 0, 2, 4, 6, and 8 days post-drought treatment for physiological characterization and qRT-PCR profiling. Three biological replicates were harvested per time point and variety. The gathered samples were placed in an ultra-low-temperature freezer set at −80 °C after being rapidly frozen in liquid nitrogen.

### 2.8. Relevant Physiological Indexes Were Measured

Malondialdehyde (MDA) content was determined using the thiobarbituric acid (TBA) method [41]. Superoxide dismutase (SOD) activity was measured using the nitroblue tetrazolium (NBT) method [42], whereas peroxidase (POD) activity was assessed using the guaiacol method [43]. The catalase (CAT) activity was determined using the ultraviolet absorption method, as described by Abei [44]. For sample preparation, 0.1 g of leaves were placed in a 2 mL centrifuge tube, frozen with liquid nitrogen after adding steel balls, and ground into powder at 1500 rpm for 30 s using a tissue grinding instrument. For MDA content measurement, 1.5 mL of 0.1% trichloroacetic acid (TCA) was added as the extraction solution. For SOD, POD, and CAT activity measurements, 1 mL of 50 mM phosphate buffer (pH 7.8) was used as the extract. The samples were shaken, mixed, and centrifuged at 12,000 rpm at 4 °C for 15 min, and the supernatant was collected as the crude enzyme solution for subsequent enzyme activity and content analysis.

### 2.9. Analysis of Expression Pattern of LOX Gene Family in Broomcorn Millet

The leaves, roots, stems, and grains of broomcorn millet seedlings frozen at low temperatures were ground into powder using a plant tissue grinding instrument. The RNaiso Plus reagent (Takara Biotechnology, Beijing, China) was used to extract total RNA from every sample. An ultra-low-volume spectrophotometer (BioDrop, Cambridge, UK) was used to measure the A260/A280 ratio and RNA concentration in order to evaluate the quality of the RNA. For reverse transcription into cDNA, samples with an A260/A280 ratio between 1.8 and 2.1 were chosen.

Reverse transcription was performed using the PrimeScript^TM^ RT Reagent Kit with gDNA Eraser (Perfect Real Time) (Takara Biotechnology, Beijing, China) following the manufacturer’s protocol, and the resulting cDNA was diluted fivefold for qRT-PCR analysis. qRT-PCR analysis was performed on a Bio-Rad CFX96 real-time fluorescence quantitative PCR system using a TB Green^®^ Premix Ex Taq^TM^ II (Tli RNaseH Plus) kit (Takara Biotechnology, Dalian, China).

The qRT-PCR protocol was configured as follows: initial denaturation at 95 °C for 30 s, followed by 40 cycles of denaturation at 95 °C for 5 s and annealing and extension at 60 °C for 30 s. A stepwise temperature transition from 60 °C to 95 °C with 0.5 °C increment for 5 s was applied to generate the melting curve. Relative gene expression levels were calculated using the ΔΔCq method using Bio-Rad Manager 3.1 software [45]. Real-time fluorescent quantitative primers were designed using Primer Premier 5 software based on the CDS sequence of the broomcorn millet *LOX* gene, with ACT2 as a drought-stress-stable gene from switchgrass used as an internal reference for broomcorn millet [31].

## 3. Results

### 3.1. Identification and Characterization of the LOX Gene Family in Broomcorn Millet

**Figure 1:**
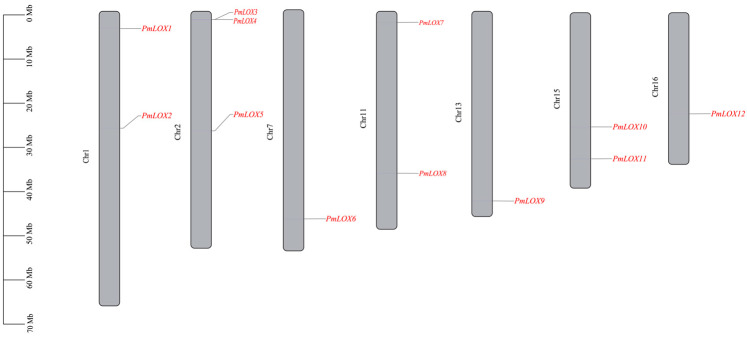
Chromosome localization of *LOX* family members of broomcorn millet.

The conserved domains of the identified proteins were confirmed by comparing their sequences with the LOX protein sequences in Arabidopsis. Sequences lacking both the lipoxygenase domain and PLAT/LH2 domain were excluded. Consequently, 12 *LOX* family members were identified in broomcorn millet and designated as *PmLOX1*-*PmLOX12* (Table 1). These genes were dispersed irregularly throughout the seven chromosomes of broomcorn millet (Figure 1). Chr2 contained the highest number of members (3), whereas Chr1, Chr11, and Chr15 each contained two members. Chr7, Chr13, and Chr16 had only one *LOX* gene each.

The physicochemical properties and subcellular localization predictions for the 12 PmLOX proteins are summarized in Table 1. The coding sequences (CDS) of *PmLOX* genes ranged from 2253 bp (*PmLOX4*) to 2856 bp (*PmLOX12*), with the corresponding protein lengths varying from 750 (PmLOX4) to 951 (PmLOX12) amino acids. The protein molecular weights ranged from 83,630.97 (PmLOX12) to 105,900.23, and the isoelectric points (pI) spanned between 5.65 (PmLOX4) and 8.49 (PmLOX9). Notably, only PmLOX1, PmLOX8, and PmLOX9 had pI values above 7, indicating that they were basic proteins, whereas the remaining nine members had pI values below 7, classifying them as acidic proteins. The average hydrophilicity coefficients of broomcorn millet LOX proteins were negative, indicating that they were hydrophilic proteins. The subcellular localization predictions revealed that PmLOX proteins were primarily localized in the cytoplasm and chloroplasts. Specifically, there were six proteins localized to the cytoplasm, including PmLOX1, PmLOX5, PmLOX6, PmLOX8, PmLOX10, and PmLOX12, and six proteins localized to the chloroplasts, including PmLOX2, PmLOX3, PmLOX4, PmLOX7, PmLOX9, and PmLOX11.

### 3.2. Systematic Evolutionary Analysis of the LOX Gene Family in Broomcorn Millet

**Figure 2:**
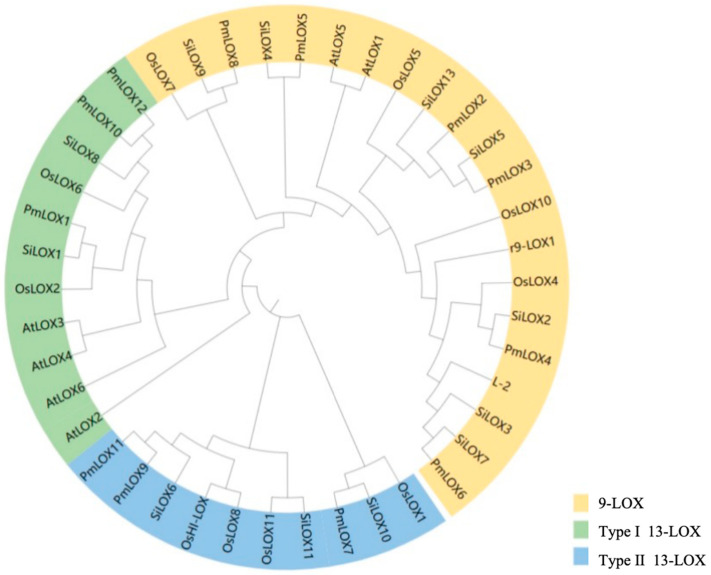
Phylogenetic tree of the *LOX* gene family in broomcorn millet.

To examine the evolutionary relationships of broomcorn millet *LOX* genes, the 12 identified broomcorn millet LOX protein sequences, along with 6 AtLOX (Arabidopsis), 12 OsLOX (*Oryza sativa*), and 12 SiLOX (*Setaria italica*) protein sequences, were aligned using MEGA7.0, and a phylogenetic tree was created (Figure 2). A phylogenetic tree was constructed to classify the *LOX* genes of broomcorn millet into three subfamilies. Six *PmLOX* members (*PmLOX2*, *PmLOX3*, *PmLOX4*, *PmLOX5*, *PmLOX6*, and *PmLOX8*) were assigned to the 9-LOX subfamily, and the remaining six members were grouped into the 13-LOX subfamily. Based on previously established *LOX* classification criteria [16], the type I 13-LOX subfamily included three *PmLOX* genes (*PmLOX1*, *PmLOX10*, and *PmLOX12*), and the type II 13-LOX subfamily included three *PmLOX* genes (*PmLOX7*, *PmLOX9*, and *PmLOX11*). Notably, type I 13-LOX members can be present only in rice, foxtail millet, and broomcorn millet and can be absent in Arabidopsis [46].

### 3.3. Protein Secondary Structure Prediction, Motif Analysis, and Conserved Domain Analysis of Members of the LOX Gene Family Members in Broomcorn Millet

Analysis of the protein sequences of the broomcorn millet *LOX* gene family revealed two characteristic domains: PLAT/LH2 and Lipoxygenase (Table 2). The members (*PmLOX1* to *PmLOX12*) contained both domains, demonstrating typical features of the *LOX* gene family.

**Figure 3:**
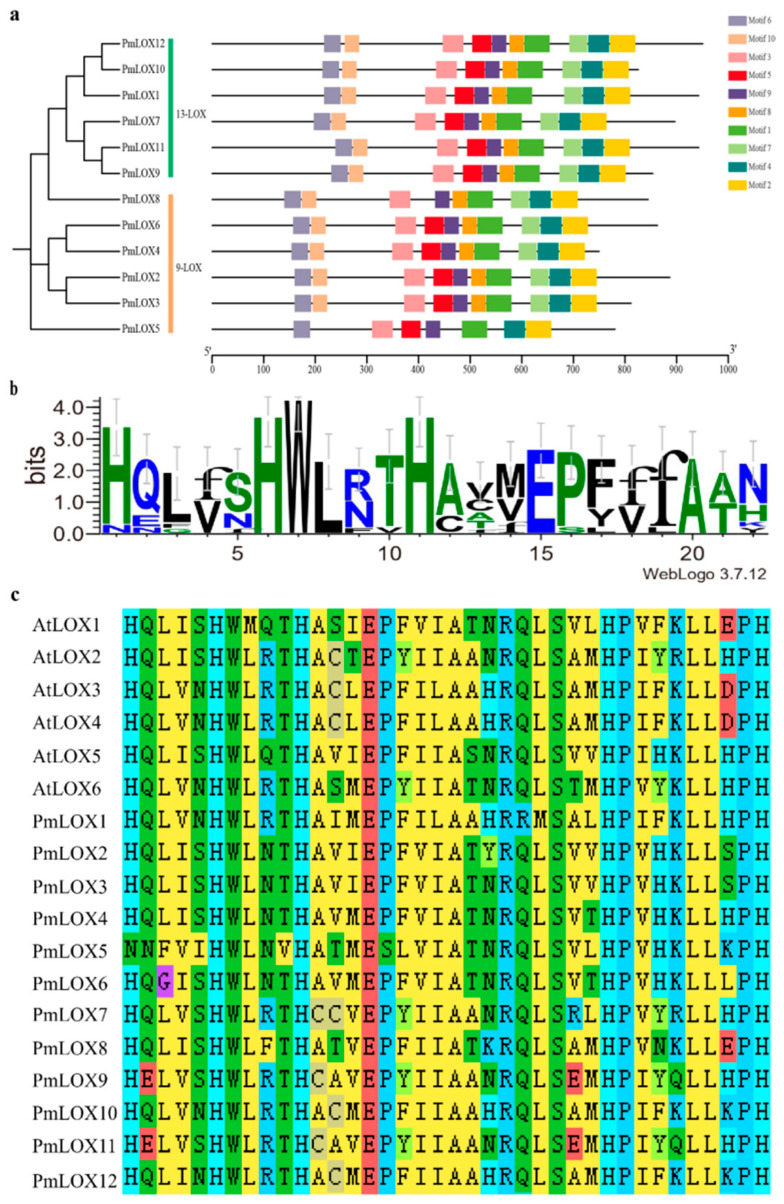
Motifs and conserved domains of the broomcorn millet *LOX* family members. (**a**) Phylogenetic tree constructed based on the protein sequences of PmLOX and motif analysis; (**b**) identification of the conserved domain sequence of 38 residues in motif 1; (**c**) alignment of the conserved sequence of 38 residues in AtLOX and PmLOX protein sequences.

Conserved motif analysis of the amino acid sequences of the 12 *PmLOX* family members identified 10 motifs. Based on the classification of *PmLOX* members in Section 3.2, all 13-LOX members were observed to contain all 10 motifs, whereas most 9-LOX members also contained nearly all motifs, except *PmLOX8*, which lacked motif 5, as well as *PmLOX5* lacking motifs 7, 8, and 10. Motif 1 contains the characteristic lipoxygenase domain of the *LOX* family, and is highly conserved across all broomcorn millet *LOX* family members, including a histidine-rich segment [His-(X)4-His-(X)4-His-(X)17-His-(X)8-His]. This domain, consisting of 38 amino acids, is important for maintaining the structural stability of LOXs. Notably, in *PmLOX5*, the first histidine (H) in this segment is replaced by asparagine (N) (Figure 3).

To further characterize PmLOX proteins, their secondary structures were predicted (Table 3). The analysis revealed that the PmLOX proteins primarily consisted of α helices, which accounted for the highest proportion, ranging from 42.04% to 46.06%. This was followed by α-helices at 34.79% to 39.87%, extending the strands at 12.62% to 14.42%, and β-turns at the lowest proportion, ranging from 3.24% to 5.73%.

### 3.4. Analysis of Cis-Acting Elements of LOX Gene Family Promoter in Broomcorn Millet

**Figure 4:**
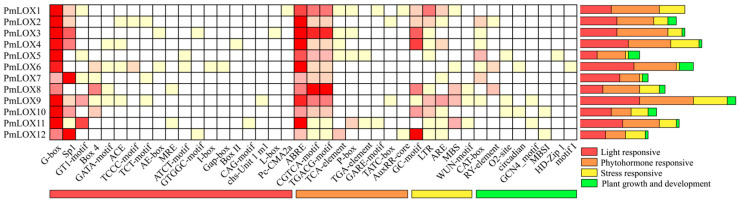
*Cis*-acting elements statistics in the upstream promoter of *LOX* gene in broomcorn millet.

To investigate the regulatory expression patterns of *PmLOX* genes in broomcorn millet, 2000 bp upstream sequences from their transcription start sites were analyzed using the PlantCARE online tool to identify the *cis*-acting elements in the promoters of the 12 *PmLOX* genes. A total of 41 *cis*-acting elements were identified, including 21 light-responsive elements, 9 hormone-responsive elements, 5 stress-responsive elements, and 6 elements related to plant growth and development (Figure 4). These findings suggest that *PmLOX* expression is regulated by multiple factors.

Analysis of the 12 *PmLOX* genes revealed that 10 contained low-temperature stress-related elements (LTR), while 9 contained tissue-specific or growth and development-responsive elements, such as CAT-box, GCN4_motif, and RY-element. Eight genes included anaerobic induction elements (ARE) and hypoxia-specific induction elements (GC-motif), while five contained MYB-binding sites involved in drought induction (MBS). Additionally, two genes had diurnal rhythm regulation elements (CAT-box and circadian), and one gene contained a mechanical injury response element (WUN-motif). All *PmLOX* genes exhibited numerous light-responsive regulatory elements, with the G-box, Sp1, and GT1-motif being the most common. Each *PmLOX* gene contains at least seven hormone-responsive *cis*-acting elements, including those related to abscisic acid (ABA) and methyl jasmonate (MeJA). ABA-responsive elements (ABREs) were the most abundant, followed by MeJA-responsive elements (CGTCA-motif- and TGACG-motifs). Some genes also included elements responsive to gibberellic acid (GARE-motif, P-box, TATC-box), auxin (AuxRR core, TGA-element), and salicylic acid (TCA-element). These findings suggest that most of the *PmLOX* genes play a role in the responses of broomcorn millet to abiotic stress.

### 3.5. Physiological Response of Broomcorn Millet Seedlings to Drought Stress at Seedling Stage

**Figure 5:**
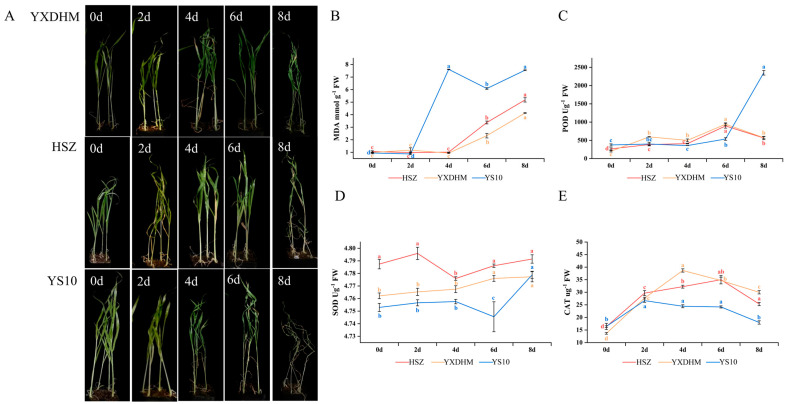
Physiological response of HSZ, YXDHM, and YS10 under drought stress. (**A**) Phenotypic changes in leaves, stems. (**B**) MDA content. (**C**) POD activity. (**D**) SOD activity. (**E**) CAT activity. Data are presented as mean ± SD (n = 3). Different lowercase letters indicate a significant difference between drought stress at different times in the same variety (*p* < 0.05). MDA: malondialdehyde; POD: peroxidase; SOD: superoxide dismutase; CAT: catalase.

Three broomcorn millet samples with varying drought tolerance were subjected to natural drought stress, and changes in physiological indices, including POD, SOD, CAT, and MDA, were measured. The results (Figure 5) indicated significant differences in the physiological response patterns among the varieties under drought stress, which correlated markedly with their respective drought tolerance levels.

Observations of the apparent morphology under drought stress revealed that the drought-tolerant varieties HSZ and YXDHM exhibited delayed wilting symptoms compared to the drought-sensitive YS10. While YS10 displayed initial leaf wilting on day 4 post-drought, HSZ and YXDHM maintained turgor until day 6 (Figure 5A). After eight days of treatment, YS10 exhibited severe dehydration, with some plants dying, whereas HSZ and YXDHM exhibited increased wilting and inhibited growth but remained viable. Phenotypic analysis confirmed that HSZ and YXDHM possessed stronger drought resistance than YS10.

Physiological indicator responses were analyzed to validate epigenetic findings (Figure 5). The MDA content in YS10 was substantially greater than that in HSZ and YXDHM, as seen in Figure 5B. During the first two days of drought stress, the MDA content of all three kinds of varieties remained similar. However, YS10 exhibited a sharp increase in MDA levels between days 2 and 4, which remained consistently high thereafter. The MDA content in HSZ and YXDHM increased steadily between days 4 and 8 of drought stress, with HSZ showing slightly higher levels than YXDHM. However, both remained consistently lower than YS10 from day 2 onward. MDA can be a key indicator of membrane lipid peroxidation and stress intensity, as drought stress can induce its accumulation, exacerbating lipid peroxidation in plant cell membranes and causing cellular damage [47,48]. These findings indicated that compared to HSZ and YXDHM, YS10 seedlings accumulated more MDA under drought stress, resulting in greater membrane damage and weaker antioxidant capacity.

Under drought stress, the activity of the antioxidant enzyme system (POD, SOD, and CAT) in plants can be enhanced to eliminate reactive oxygen free radicals, reduce cellular damage, and increase drought resistance [49,50,51]. As shown in Figure 5C, the POD activity of the drought-resistant varieties HSZ and YXDHM initially increased and then declined, with levels higher than those of the sensitive variety YS10 on days 4 and 6. Between days 2 and 6, YXDHM displayed higher POD activity than HSZ. In contrast, YS10 showed minimal changes in POD activity in the early stages, with a significant increase after 6–8 d of drought treatment. Figure 5D illustrates that the SOD activities of HSZ and YXDHM were significantly higher than that of YS10, whereas the activity in HSZ initially decreased before increasing. YXDHM showed a continuous increase. In YS10, SOD activity mirrored the trend in POD activity, presenting a marked increase after 6–8 d of drought treatment. As shown in Figure 5E, CAT activity of the three experimental varieties initially increased and then decreased under drought stress. YS10 exhibited significantly lower CAT activity than HSZ and YXDHM. These results were consistent with the observed morphological changes under drought stress, confirming that HSZ and YXDHM possess stronger drought resistance than YS10.

### 3.6. Organ-Specific Expression of PmLOX Family Members

**Figure 6:**
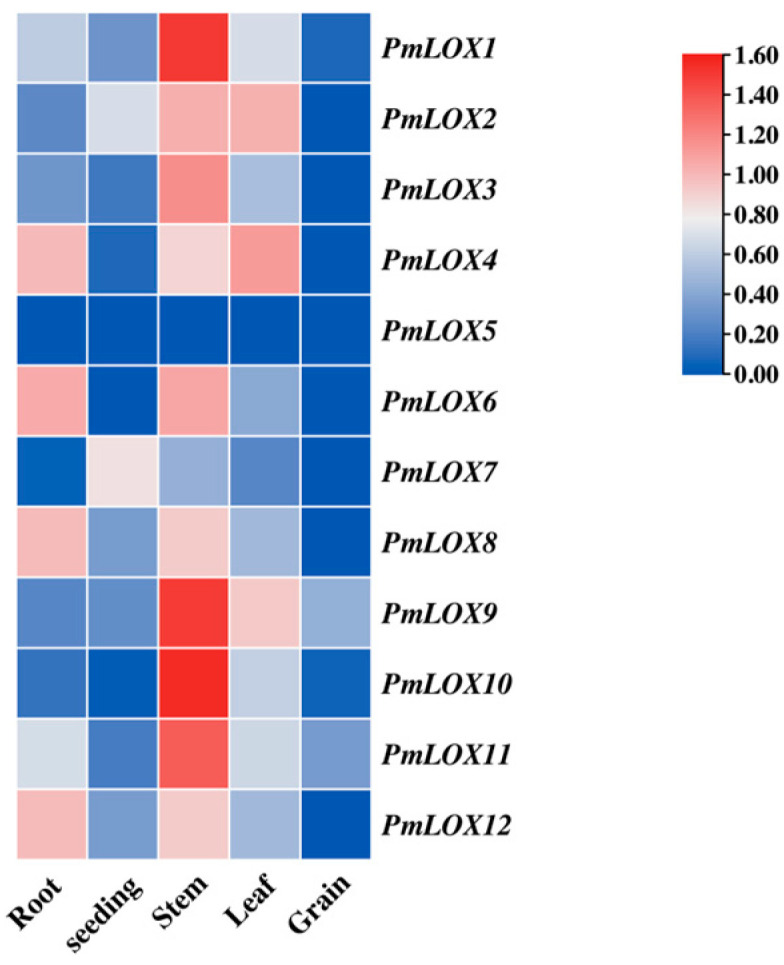
Organ-specific expression of *LOX* family in broomcorn millet.

Organ-specific expression analysis (Figure 6) revealed that *PmLOX5* was not detected in the roots, stems, leaves, seedlings, or grains. This suggests that *PmLOX5* may be expressed only at specific developmental stages or exhibit extremely low expression levels. The expression levels of the majority of broomcorn millet *LOX* genes were higher in stems than in other organs, with notably low expression in seedlings and grains. However, *PmLOX4*, *PmLOX6*, *PmLOX8*, and *PmLOX12* exhibited higher expression levels in roots than the other *LOX* genes.

### 3.7. Expression Analysis of PmLOX Family Members in Leaves of Five-Leaf-Stage Seedlings Under Drought Stress

**Figure 7:**
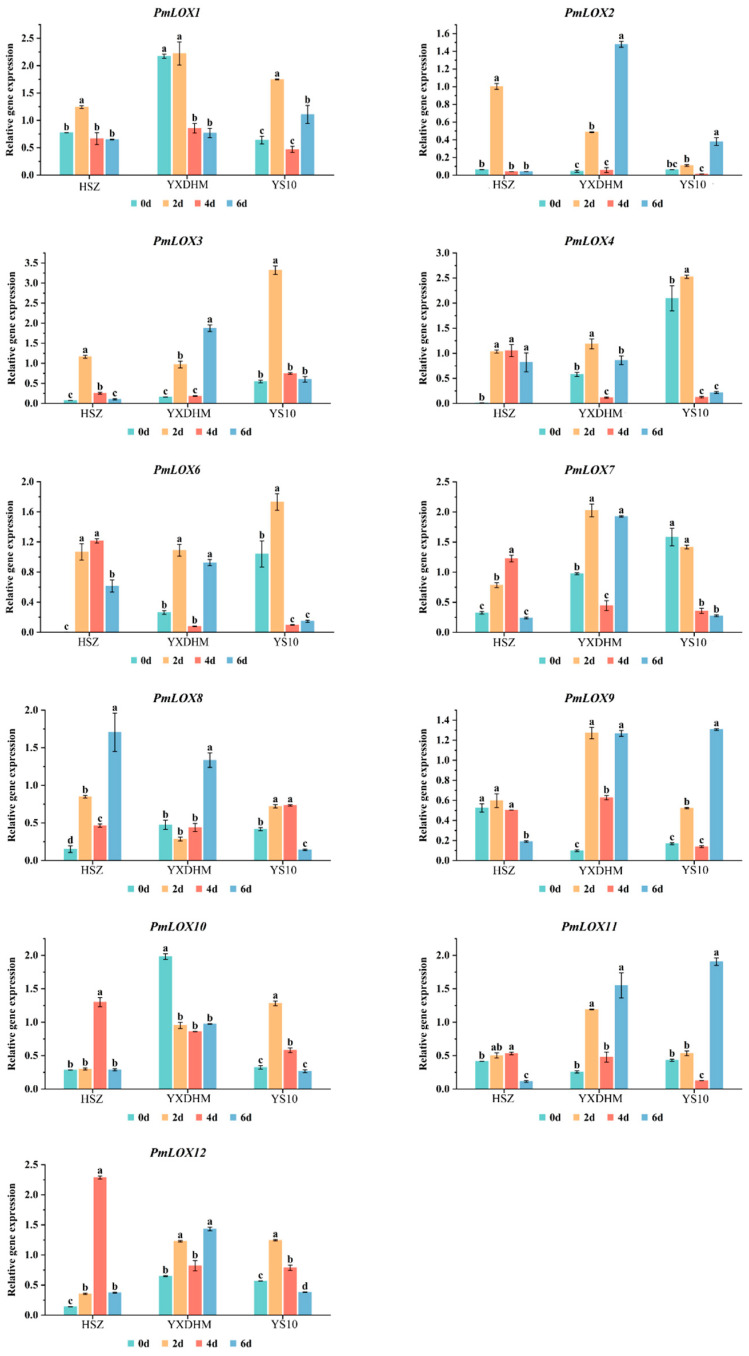
qRT-PCR analysis of the expression patterns of *LOX* genes family members in broomcorn millet under drought stress. Data are presented as mean ± SD (n = 3). Different lowercase letters indicate significant differences (*p* < 0.05) based on one-way ANOVA with Tukey’s post hoc test.

To explore the role of *LOX* gene family members in drought stress in broomcorn millet, qRT-PCR was used to analyze their expression patterns across the varieties of three broomcorn millet (Figure 7). Among the 12 *PmLOX* genes, *PmLOX5* was not expressed at any of the four drought sampling time points. The remaining 11 *PmLOX* genes responded to the drought stress. *PmLOX1* and *PmLOX10* in HSZ and YS10 exhibited similar expression patterns, with the levels initially increasing and then decreasing as drought duration progressed. In YXDHM, the expression of *PmLOX1* and *PmLOX10* was downregulated under drought stress, with *PmLOX1* remaining low after 4 d and *PmLOX10* after 2 d. *PmLOX6* showed the highest expression in drought-sensitive variety YS10, whereas its expression decreased significantly after 4 d of drought. In YXDHM, *PmLOX7*, *PmLOX9*, and *PmLOX11* displayed an “up–down–up” expression pattern under prolonged drought stress, whereas in YS10, *PmLOX9* and *PmLOX11* followed a similar trend, peaking on day 6. However, *PmLOX7* was inhibited and its expression declined as drought stress intensified.

Compared to YXDHM and YS10, HSZ had substantially decreased expression levels of *PmLOX7*, *PmLOX9*, and *PmLOX11*. Under drought conditions, *PmLOX9* was downregulated, although the other two genes’ expression levels first rose and subsequently fell. In the drought-sensitive variety YS10, *PmLOX3* and *PmLOX4* expression levels were noticeably greater in the drought-tolerant varieties HSZ and YXDHM, peaking at day 2 of drought treatment. Conversely, *PmLOX8* in drought-tolerant plants displayed an overall upward trend, with expression levels higher than those in YS10.

The expression of *PmLOX2* in the drought-tolerant varieties HSZ and YXDHM followed distinct patterns, showing a “first increase and then decrease” trend in HSZ and an “increase–decrease–increase” trend in YXDHM, with peaks at day 2 and day 6 under drought stress, respectively. The expression levels were 2.6 and 3.8 times higher than those in drought-sensitive variety YS10. The *PmLOX12* expression in YXDHM and YS10 was similar, whereas in HSZ, *PmLOX12* gradually increased, peaking at day 4, significantly exceeding the levels in YS10. *PmLOX8* in the drought-tolerant plants exhibited an overall upward trend, with expression levels higher than those in YS10. A comprehensive analysis of the correlation between *LOX* gene expression and physiological indices under drought stress suggests that *PmLOX2*, *PmLOX12*, and *PmLOX8* are probably involved in the drought stress response of broomcorn millet.

## 4. Discussion

LOXs are key enzymes in the fatty acid metabolic pathway of plants and play a important role in growth, development, and stress responses. The *LOX* gene family has been extensively studied in various crops, with 6 LOXs identified in Arabidopsis [23], 14 in rice [23], 12 in foxtail millet [24], 14 in tomato [25], 18 in grape [26], and 23 in cucumber [52]. However, the *LOX* gene family in broomcorn millet is largely unknown. Based on the six *LOX* members found in Arabidopsis, twelve members of the *LOX* gene family were identified in the genome of broomcorn millet in this study. According to chromosome localization studies, these genes were dispersed irregularly across seven chromosomes, with a total count double that of Arabidopsis, potentially reflecting the polyploidy and evolutionary history of broomcorn millet.

Phylogenetic tree analysis classified the broomcorn millet *LOX* gene family into three subfamilies, which is consistent with the findings of Feussner and Wasternack [16]. *PmLOX2*, *PmLOX3*, *PmLOX4*, *PmLOX5*, *PmLOX6*, and *PmLOX8* are assigned to the 9-LOX subfamily. The six 13-LOX members were further divided into type I and type II 13-LOX subfamilies, based on the presence of a chloroplast transit peptide in the N-terminal sequence of type II LOX proteins. *PmLOX1*, *PmLOX10*, and *PmLOX12* are classified as type I 13-LOX, whereas *PmLOX7*, *PmLOX9*, and *PmLOX11* are classified as type II 13-LOX. Notably, type I 13-LOX subfamily members were identified exclusively in rice, foxtail millet, and broomcorn millet and were absent in Arabidopsis, consistent with the classification results for Arabidopsis and rice [23,46].

Conserved motif analysis of broomcorn millet LOX proteins identified 10 motifs, most of which were highly conserved across the *PmLOX* members. However, *PmLOX8* lacked motif 5, and *PmLOX5* lacked motifs 7, 8, and 10, suggesting that these proteins may have additional or unique functions. All broomcorn millet LOX proteins contain a highly conserved lipoxygenase domain within motif 1, composed of 38 amino acid residues, consistent with the findings in foxtail millet and cotton [24,53]. Notably, in *PmLOX5*, the first histidine (H) in the lipoxygenase domain was replaced with asparagine (N) (Figure 3b,c), coinciding with the absence of conserved motifs. To further understand the regulatory expression patterns of *PmLOX* genes under various conditions, their promoter cis-acting elements were predicted and analyzed, revealing four categories: light response, hormone response, stress response, and plant growth and development. These elements include those related to abscisic acid, MeJA, low-temperature stress, drought induction, and tissue-specific expression. This diversity of *cis*-acting elements aligned with the studies highlighting the functional versatility of plant *LOX* genes [54,55]. Notably, MeJA, a hormone involved in plant signal transduction, has been extensively explored in crops such as wheat [56], rice [57], pepper [58], and tomato [59]. By inducing the expression of defense-related genes, research has demonstrated significant positive effects of MeJA on plant resistance to biotic stresses, such as pest infestations, and abiotic stresses like drought and low temperatures [60]. In broomcorn millet, all *LOX* genes were found to contain the MeJA-responsive CGTCA-motif and TGACG-motif, suggesting that the *PmLOX* gene family may enhance plant resistance to both abiotic and biotic stresses.

To investigate the potential functions of *LOX* family members in broomcorn millet growth and development, three kinds of varieties with varying levels of drought resistance were subjected to drought stress, and their roles were analyzed by integrating physiological indices and *PmLOX* gene expression patterns. ROS in plants are maintained in a dynamic balance under typical environmental circumstances. However, drought stress disrupts this balance, which leads to disordered ROS production and metabolism. ROS-mediated oxidative stress induces harmful cellular effects, including lipid peroxidation of biological membranes and obstruction of photosynthesis [47,48,49,50,51]. As a product of membrane lipid peroxidation, MDA can severely damage the biofilm system, and its accumulation can indicate the extent of plant injury [47,48]. In response to drought stress, the activity of the antioxidant enzyme system (POD, SOD, and CAT) increases to remove ROS, mitigate plant damage, and enhance drought resistance [49,50,51]. In this study, HSZ and YXDHM exhibited lower MDA levels than YS10, indicating a stronger antioxidant activity and reduced cellular damage. Additionally, the activities of SOD, POD, and CAT were significantly higher in HSZ and YXDHM than in YS10, further confirming their superior drought resistance (Figure 5).

Organ-specific expression analysis of *PmLOX* genes demonstrated that most *LOX* genes were not expressed in grains, while all were expressed in other organs, with particularly high expression levels in stems. Notably, *PmLOX5* was not expressed in the roots, stems, leaves, seedlings, or grains, consistent with its absence under drought stress. This suggests that *PmLOX5* may exhibit extremely low expression levels or be expressed exclusively during specific developmental stages, which requires further experimental validation.

Expression pattern analysis revealed that most *LOX* genes in broomcorn millet exhibited increased expression levels under drought stress, which was consistent with the *LOX* response to drought stress observed in cotton, radish, and foxtail millet [24,53,61]. Broomcorn millet employs various tolerance mechanisms to mitigate abiotic stresses. Under drought conditions, most *PmLOX* genes were upregulated at different expression levels. Notably, *PmLOX1* and *PmLOX10* were downregulated in YXDHM, whereas *PmLOX3*, *PmLOX4*, *PmLOX9*, and *PmLOX11* demonstrated higher expression levels in the drought-sensitive variety YS10 than in the drought-tolerant varieties HSZ and YXDHM. Compared to other *LOX* genes, *PmLOX4*, *PmLOX6*, *PmLOX8*, and *PmLOX12* have greater expression levels in the roots.

In foxtail millet, Zhang et al. [24] identified 12 *LOX* genes and demonstrated that *SiLOX7* was significantly upregulated under drought stress, particularly in drought-resistant varieties. Similarly, our study identified 12 *LOX* genes in broomcorn millet, with *PmLOX2* showing marked upregulation in drought-tolerant genotypes. However, unlike *SiLOX7* with root-specific expression, *PmLOX2* exhibited higher expression levels in both leaves and stems, suggesting tissue-specific functional diversification between these two closely related species. This divergence highlights the potential for species-specific adaptation mechanisms within the *LOX* gene family.

Previous studies have indicated that *AtLOX6* can be significant for the buildup of jasmonic acid (JA) in Arabidopsis roots under drought stress, significantly contributing to the plant response to abiotic and biotic stresses [60]. Under drought stress, the expression levels of *PmLOX8* and *PmLOX12* in drought-resistant plants were slightly higher than those in YS10, suggesting that their role in the drought stress response of broomcorn millet may primarily involve the accumulation of root derivatives. *PmLOX2*, a member of the 9-LOX subfamily, was upregulated in the drought-resistant varieties HSZ and YXDHM, whereas its expression in YS10 remained relatively unchanged. *PmLOX2* is homologous to *AtLOX1* and *AtLOX5* in Arabidopsis. Previous studies have shown that the Arabidopsis mutants *lox1* and *lox5* exhibited increased membrane lipid peroxidation, higher MDA accumulation, and reduced stress resistance compared to the wild type [62]. In this study, HSZ and YXDHM, which exhibited high *PmLOX2* expression, had significantly higher SOD and CAT activities and lower MDA accumulation than YS10, which is consistent with previous findings [62]. The increased activity of antioxidant enzymes (POD, SOD, and CAT) in the drought-resistant varieties HSZ and YXDHM resulted in lower MDA accumulation and reduced cellular oxidation. This response is likely linked to the upregulation of *LOX* genes, including *PmLOX2*, which correlates with improved drought adaptation and reduced cellular damage.

Our study identified 12 *LOX* gene family members in broomcorn millet, classified into three subfamilies, and revealed their potential roles in drought tolerance. Notably, *PmLOX2* was strongly induced in drought-tolerant varieties, correlating with enhanced antioxidant capacity and reduced oxidative damage.

However, our study has some limitations. The absence of growth and yield data under drought conditions restricts direct correlations between *PmLOX* expression and physiological responses. Subcellular localizations predictions require experimental validation. Additionally, the use of a single reference gene (ACT2) may introduce technical bias; future studies should incorporate multiple reference genes to enhance reliability. To advance these findings, functional validation of *PmLOX2* via CRISPR/Cas9 knockout and its interaction with jasmonate signaling under drought should be prioritized. Such data would elucidate the mechanistic role of *LOX* genes in drought adaptation and their potential for improving stress-resilient crops.

## 5. Conclusions

This study identified 12 *LOX* gene family members in broomcorn millet using bioinformatic methods and categorized them into three subfamilies, each exhibiting distinct structural features and conserved motifs, and were unevenly distributed across seven chromosomes. Promoter analysis revealed the presence of stress-responsive cis-elements, such as ABA, MeJA, and drought-inducible motifs, suggesting their potential involvement in stress signaling pathways. Organ-specific expression profiles indicated that most *PmLOX* genes were predominantly expressed in stems, while *PmLOX5* showed no detectable expression, implying its potential role in specific developmental stages or extremely low expression levels. The response of *PmLOX* genes to drought stress was examined, revealing notable similarities and specificities in gene expression patterns among different drought-resistant varieties in broomcorn millet. Notably, *PmLOX2* expression correlated with improved drought adaptation, suggesting its potential role in regulating ROS scavenging and membrane protection mechanisms. These findings establish a basis for further exploration of the functional role of *LOX* in broomcorn millet.

## Figures and Tables

**Table 1:** Physicochemical properties and subcellular localization predictions of *LOX* family members in broomcorn millet.

Gene Name	Gene ID	CDS Length	Proteinsize	Molecule Weight	PI	Subcellular Localization	Signal Peptide	Instability Index	GRAVY
*PmLOX1*	RLN40792.1	2832	943	104,995.76	8.16	Cytoplasmic		47.17	−0.343
*PmLOX2*	RLN40543.1	2664	887	100,422.94	6.12	Chloroplast	Chloroplast Transit peptide	42.59	−0.401
*PmLOX3*	RLN18482.1	2439	812	91,653.09	6.09	Chloroplast	Chloroplast Transit peptide	40.45	−0.387
*PmLOX4*	RLN16584.1	2253	750	83,630.97	5.65	Chloroplast	Chloroplast Transit peptide	33.64	−0.277
*PmLOX5*	RLN17039.1	2346	781	86,394.9	6.42	Cytoplasmic		45.39	−0.319
*PmLOX6*	RLN23364.1	2592	863	97,311.53	5.81	Cytoplasmic		36.66	−0.33
*PmLOX7*	RLN07160.1	2694	897	101,490.55	6.18	Chloroplast	Chloroplast Transit peptide	44.45	−0.414
*PmLOX8*	RLN09190.1	2538	845	95,801.33	7.17	Cytoplasmic		48.87	−0.403
*PmLOX9*	RLN03699.1	2565	854	94,241.03	8.49	Chloroplast	Chloroplast Transit peptide	50.73	−0.361
*PmLOX10*	RLM74388.1	2481	826	92,480.33	6.66	Cytoplasmic		51.99	−0.424
*PmLOX11*	RLM74184.1	2832	943	103,347.08	6.52	Chloroplast	Chloroplast Transit peptide	50.21	−0.325
*PmLOX12*	RLM64776.1	2856	951	105,900.23	6.36	Cytoplasmic		51.8	−0.418

**Table 2:** Conserved domains of *LOX* family members in broomcorn millet.

Name	ID	PLAT/LH2 (IPR001024)	Lipoxygenase (IPR013819)
*PmLOX1*	RLN40792.1	83-223	669-926
*PmLOX2*	RLN40543.1	21-166	177-863
*PmLOX3*	RLN18482.1	21-166	177-772
*PmLOX4*	RLN16584.1	16-160	171-748
*PmLOX5*	RLN17039.1	40-164	270-543, 567-764
*PmLOX6*	RLN23364.1	19-163	174-842
*PmLOX7*	RLN07160.1	72-203	214-880
*PmLOX8*	RLN09190.1	17-146	157-822
*PmLOX9*	RLN03699.1	96-237	248-849
*PmLOX10*	RLM74388.1	81-220	231-825
*PmLOX11*	RLM74184.1	103-245	256-926
*PmLOX12*	RLM64776.1	84-223	234-934

**Table 3:** Secondary structure of the LOX protein in broomcorn millet.

Name	ID	α-Helix	β-Turn	Extended Strand	Random Coil
*PmLOX1*	RLN40792.1	39.87%	4.88%	12.62%	42.63%
*PmLOX2*	RLN40543.1	37.20%	5.52%	13.08%	44.19%
*PmLOX3*	RLN16584.1	37.07%	5.47%	13.07%	44.40%
*PmLOX4*	RLN15477.1	37.95%	3.24%	14.39%	44.42%
*PmLOX5*	RLN18482.1	37.44%	5.67%	13.55%	43.35%
*PmLOX6*	RLN23364.1	36.73%	5.45%	14.14%	43.68%
*PmLOX7*	RLN07160.1	39.35%	5.35%	13.15%	42.14%
*PmLOX8*	RLN09190.1	34.79%	5.56%	13.85%	45.80%
*PmLOX9*	RLN03699.1	39.81%	5.04%	13.11%	42.04%
*PmLOX10*	RLM74388.1	35.84%	5.69%	13.80%	44.67%
*PmLOX11*	RLM74184.1	37.43%	5.73%	14.42%	42.42%
*PmLOX12*	RLM64776.1	36.49%	4.73%	12.72%	46.06%

## Data Availability

All data generated or analyzed during this study are included in this published article.

## Abbreviations

The following abbreviations are used in this manuscript:

LOXs	Lipoxygenases
SOD	Superoxide Dismutase
POD	Peroxidase
CAT	Catalase
MDA	Malondialdehyde
ABA	Abscisic Acid
MeJA	Methyl Jasmonate
JA	Jasmonic Acid
*SWC*	Soil Water Content

## References

[1] Wahab A., Abdi G., Saleem M.H., Ali B., Ullah S., Shah W., Mumtaz S., Yasin G., Muresan C.C., Marc R.A. (2022). Plants’ Physio-Biochemical and Phyto-Hormonal Responses to Alleviate the Adverse Effects of Drought Stress: A Comprehensive Review. Plants.

[2] Khatun M., Sarkar S., Era F.M., Islam A.K.M.M., Anwar M.P., Fahad S., Datta R., Islam A.K.M.A. (2021). Drought Stress in Grain Legumes: Effects, Tolerance Mechanisms and Management. Agronomy.

[3] Farooq M., Wahid A., Kobayashi N., Fujita D., Basra S.M.A., Lichtfouse E., Navarrete M., Debaeke P., Véronique S., Alberola C. (2009). Plant Drought Stress: Effects, Mechanisms and Management. Sustainable Agriculture.

[4] Alam H., Khattak J.Z.K., Ksiksi T.S., Saleem M.H., Fahad S., Sohail H., Ali Q., Zamin M., El-Esawi M.A., Saud S. (2021). Negative impact of long-term exposure of salinity and drought stress on native *Tetraena mandavillei* L.. Physiol. Plant.

[5] Lu H., Zhang J., Liu K.B., Wu N., Li Y., Zhou K., Ye M., Zhang T., Zhang H., Yang X. (2009). Earliest domestication of common millet (*Panicum miliaceum*) in East Asia extended to 10,000 years ago. Proc. Natl. Acad. Sci. USA.

[6] Zhang Y., Han H., Zhang D., Li J., Gong X., Feng B., Xue Z., Yang P. (2017). Effects of ridging and mulching combined practices on proso millet growth and yield in semi-arid regions of China. Field Crops Res..

[7] Wang R., Hunt H.V., Qiao Z., Wang L., Han Y. (2016). Diversity and Cultivation of Broomcorn Millet (*Panicum miliaceum* L.) in China: A Review. Econ. Bot..

[8] Gong X., Dang K., Lv S., Zhao G., Tian L., Luo Y., Feng B. (2020). Interspecific root interactions and water-use efficiency of intercropped proso millet and mung bean. Eur. J. Agron..

[9] Singh P., Arif Y., Miszczuk E., Bajguz A., Hayat S. (2022). Specific Roles of Lipoxygenases in Development and Responses to Stress in Plants. Plants.

[10] Upadhyay R.K., Mattoo A.K. (2018). Genome-wide identification of tomato (*Solanum lycopersicum* L.) lipoxygenases coupled with expression profiles during plant development and in response to methyl-jasmonate and wounding. J. Plant Physiol..

[11] Laczko R., Csiszar K. (2020). Lysyl Oxidase (LOX): Functional Contributions to Signaling Pathways. Biomolecules.

[12] Yang S., Li D., Li S., Yang H., Zhao Z. (2022). GC-MS Metabolite and Transcriptome Analyses Reveal the Differences of Volatile Synthesis and Gene Expression Profiling between Two Apple Varieties. Int. J. Mol. Sci..

[13] Mostafa S., Wang Y., Zeng W., Jin B. (2022). Floral Scents and Fruit Aromas: Functions, Compositions, Biosynthesis, and Regulation. Front. Plant Sci..

[14] Lu H., Li L., Xu Y., Li D., Li G., Yan Y., Wu Q., Luo Z. (2022). FaLEC2 repressing FaLOX2 promoter involved in the metabolism of LOX-derived volatiles during strawberry ripening. Sci. Hortic..

[15] Camargo P.O., Calzado N.F., Budzinski I.G.F., Domingues D.S. (2023). Genome-Wide Analysis of Lipoxygenase (LOX) Genes in Angiosperms. Plants.

[16] Feussner I., Wasternack C. (2022). The lipoxygenase pathway. Annu. Rev. Plant Biol..

[17] Mou Y., Sun Q., Yuan C., Zhao X., Wang J., Yan C., Li C., Shan S. (2022). Identification of the LOX Gene Family in Peanut and Functional Characterization of AhLOX29 in Drought Tolerance. Front. Plant Sci..

[18] Schaller F., Schaller A., Stintzi A. (2004). Biosynthesis and Metabolism of Jasmonates. J. Plant Growth Regul..

[19] Matsui K., Koeduka T. (2016). Green Leaf Volatiles in Plant Signaling and Response. Sub-Cell. Biochem..

[20] Prost I., Dhondt S., Rothe G., Vicente J., Rodriguez M.J., Kift N., Carbonne F., Griffiths G., Esquerré-Tugayé M.T., Rosahl S. (2005). Evaluation of the antimicrobial activities of plant oxylipins supports their involvement in defense against pathogens. Plant Physiol..

[21] Vogt J., Schiller D., Ulrich D., Schwab W., Dunemann F. (2013). Identification of lipoxygenase (LOX) genes putatively involved in fruit flavour formation in apple (*Malus* × *domestica*). Tree Genet. Genomes.

[22] Meng Y., Liang Y., Liao B., He W., Liu Q., Shen X., Xu J., Chen S. (2022). Genome-Wide Identification, Characterization and Expression Analysis of Lipoxygenase Gene Family in *Artemisia annua* L.. Plants.

[23] Umate P. (2011). Genome-wide analysis of lipoxygenase gene family in Arabidopsis and rice. Plant Signal. Behav..

[24] Zhang Q., Zhao Y., Zhang J., Li X., Ma F., Duan M., Zhang B., Li H. (2021). The Responses of the Lipoxygenase Gene Family to Salt and Drought Stress in Foxtail Millet (*Setaria italica*). Life.

[25] Upadhyay R.K., Handa A.K., Mattoo A.K. (2019). Transcript Abundance Patterns of 9- and 13-Lipoxygenase Subfamily Gene Members in Response to Abiotic Stresses (Heat, Cold, Drought or Salt) in Tomato (*Solanum lycopersicum* L.) Highlights Member-Specific Dynamics Relevant to Each Stress. Genes.

[26] Andriy P., Jackie W., Brian J., Chris W. (2010). Identification of the lipoxygenase gene family from *Vitis vinifera* and biochemical characterisation of two 13-lipoxygenases expressed in grape berries of Sauvignon Blanc. Funct. Plant Biol..

[27] Kaur D., Dorion S., Jmii S., Cappadocia L., Bede J.C., Rivoal J. (2023). Pseudophosphorylation of Arabidopsis jasmonate biosynthesis enzyme lipoxygenase 2 via mutation of Ser(600) inhibits enzyme activity. J. Biol. Chem..

[28] Vellosillo T., Martínez M., López M.A., Vicente J., Cascón T., Dolan L., Hamberg M., Castresana C. (2007). Oxylipins produced by the 9-lipoxygenase pathway in Arabidopsis regulate lateral root development and defense responses through a specific signaling cascade. Plant Cell.

[29] Huang J., Cai M., Long Q., Liu L., Lin Q., Jiang L., Chen S., Wan J. (2014). OsLOX2, a rice type I lipoxygenase, confers opposite effects on seed germination and longevity. Transgenic Res..

[30] Pan W., Liu T., He J., Dong K., Ren R., Zhang L., Yang T. (2022). Genome-wide Identification and Expression Characteristics of the YABBY Gene Family under Hypertonic Solution Stress in Broomcorn Millet (*Panicum miliaceum* L.). Genom. Appl. Biol..

[31] Cao X.N., Shen L.H., Song J., Wang J.J., Wang H.G., Chen L., Pei Y.X., Liu S.C., Qiao Z.J. (2021). Analysis of cloned sequences and expression of ASR gene family in millet. J. Anim. Plant Sci..

[32] Wang M., Liu T., He J., Dong K., Ren R., Zhang L., Yang T. (2022). Genome-wide identification of bZIP gene family in broomcorn millet and analysis of its expression characteristics under polyethylene glycol treatment in seedling stage. Chin. J. Appl. Environ. Biol..

[33] Shan Z., Jiang Y., Li H., Guo J., Dong M., Zhang J., Liu G. (2020). Genome-wide analysis of the NAC transcription factor family in broomcorn millet (*Panicum miliaceum* L.) and expression analysis under drought stress. BMC Genom..

[34] Zou C., Li L., Miki D., Li D., Tang Q., Xiao L., Rajput S., Deng P., Peng L., Jia W. (2019). The genome of broomcorn millet. Nat. Commun..

[35] Huala E., Dickerman A.W., Garcia-Hernandez M., Weems D., Reiser L., LaFond F., Hanley D., Kiphart D., Zhuang M., Huang W. (2001). The Arabidopsis Information Resource (TAIR): A comprehensive database and web-based information retrieval, analysis, and visualization system for a model plant. Nucleic Acids Res..

[36] Goodstein D.M., Shu S., Howson R., Neupane R., Hayes R.D., Fazo J., Mitros T., Dirks W., Hellsten U., Putnam N. (2012). Phytozome: A comparative platform for green plant genomics. Nucleic Acids Res..

[37] Chen C., Wu Y., Li J., Wang X., Zeng Z., Xu J., Liu Y., Feng J., Chen H., He Y. (2023). TBtools-II: A “one for all, all for one” bioinformatics platform for biological big-data mining. Mol. Plant.

[38] Gasteiger E., Gattiker A., Hoogland C., Ivanyi I., Appel R.D., Bairoch A. (2003). ExPASy: The proteomics server for in-depth protein knowledge and analysis. Nucleic Acids Res..

[39] Bailey T.L., Williams N., Misleh C., Li W.W. (2006). MEME: Discovering and analyzing DNA and protein sequence motifs. Nucleic Acids Res..

[40] Zhang M., Yang W., Qiao Z., Feng M., Wang G., Duan Y., Chen L. (2013). Resistance Evaluation and Response of 16 Millet Varieties at Germination Stage to Drought Stress. Acta Agrestia Sin..

[41] Zhang J., Kirkham M.B. (1996). Antioxidant responses to drought in sunflower and sorghum seedlings. New Phytol..

[42] Beauchamp A., Fridovich I. (1971). Superoxide dismutase: Improved assays and an assay applicable to acrylamide gels. Anal. Biochem..

[43] Sun L., Xu X., Jiang Y., Zhu Q., Yang F., Zhou J., Yang Y., Huang Z., Li A., Chen L. (2015). Genetic Diversity, Rather than Cultivar Type, Determines Relative Grain Cd Accumulation in Hybrid Rice. Front. Plant Sci..

[44] Abei H. (1984). Catalase in vitro. Methods Enzymol..

[45] Noguchi A., Nakamura K., Sakata K., Sato-Fukuda N., Ishigaki T., Mano J., Takabatak R., Kitta K., Teshima R., Kondo K. (2015). Development and Interlaboratory Validation of a Simple Screening Method for Genetically Modified Maize Using a ΔΔC(q)-Based Multiplex Real-Time PCR Assay. Anal. Chem..

[46] Bannenberg G., Martínez M., Hamberg M., Castresana C. (2009). Diversity of the enzymatic activity in the lipoxygenase gene family of *Arabidopsis thaliana*. Lipids.

[47] Li C., Chen S., Wang Y. (2023). Physiological and proteomic changes of *Castanopsis fissa* in response to drought stress. Sci. Rep..

[48] Ru C., Hu X., Chen D., Wang W., Zhen J. (2023). Photosynthetic, antioxidant activities, and osmoregulatory responses in winter wheat differ during the stress and recovery periods under heat, drought, and combined stress. Plant Sci..

[49] Nadarajah K.K. (2020). ROS Homeostasis in Abiotic Stress Tolerance in Plants. Int. J. Mol. Sci..

[50] Bandurska H. (2022). Drought Stress Responses: Coping Strategy and Resistance. Plants.

[51] Sarma B., Kashtoh H., Lama Tamang T., Bhattacharyya P.N., Mohanta Y.K., Baek K.-H. (2023). Abiotic Stress in Rice: Visiting the Physiological Response and Its Tolerance Mechanisms. Plants.

[52] Liu S.Q., Liu X.H., Jiang L.W. (2011). Genome-wide identification, phylogeny and expression analysis of the lipoxygenase gene family in cucumber. Genet. Mol. Res..

[53] Shaban M., Ahmed M., Sun H., Ullah A., Zhu L. (2018). Genome-wide identification of lipoxygenase gene family in cotton and functional characterization in response to abiotic stresses. BMC Genom..

[54] Porta H., Rocha-Sosa M. (2002). Plant lipoxygenases. Physiological and molecular features. Plant Physiol..

[55] Viswanath K.K., Varakumar P., Reddy R., Basha S.J., Ampasala A. (2020). Plant Lipoxygenases and Their Role in Plant Physiology. J. Plant Biol..

[56] Ma C., Wang Z., Sun M. (2015). Hydrogen peroxide acts as a signaling molecule for the methyl jasmonate-induced antioxidant defense in wheat callus to promote enhanced drought tolerance. J. Agric. Sci..

[57] Liu Z., Zhang S., Sun N., Liu H., Zhao Y., Liang Y., Zhang L., Han Y. (2015). Functional diversity of jasmonates in rice. Rice.

[58] Sarde S.J., Kumar A., Remme R.N., Dicke M. (2018). Genome-wide identification, classification and expression of lipoxygenase gene family in pepper. Plant Mol. Biol..

[59] Yu M., Shen L., Fan B., Zhao D., Zheng Y., Sheng J. (2009). The effect of MeJA on ethylene biosynthesis and induced disease resistance to *Botrytis cinerea* in tomato. Postharvest Biol. Technol..

[60] Yu X., Zhang W., Zhang Y., Zhang X., Lang D., Zhang X. (2019). The roles of methyl jasmonate to stress in plants. Funct. Plant Biol..

[61] Wang J., Hu T., Wang W., Hu H., Wei Q., Wei X., Bao C. (2019). Bioinformatics Analysis of the Lipoxygenase Gene Family in Radish (*Raphanus sativus*) and Functional Characterization in Response to Abiotic and Biotic Stresses. Int. J. Mol. Sci..

[62] López M.A., Vicente J., Kulasekaran S., Vellosillo T., Martínez M., Irigoyen M.L., Cascón T., Bannenberg G., Hamberg M., Castresana C. (2011). Antagonistic role of 9-lipoxygenase-derived oxylipins and ethylene in the control of oxidative stress, lipid peroxidation and plant defence. Plant J..

